# Siblings’ Perceptions of Their ADHD-Diagnosed Sibling’s Impact on the Family System

**DOI:** 10.3390/ijerph13090910

**Published:** 2016-09-13

**Authors:** Kerry King, Daleen Alexander, Joseph Seabi

**Affiliations:** Department of Psychology, University of the Witwatersrand, Private Bag 3, WITS, Gauteng 2050, South Africa; kerryleighking@gmail.com (K.K.); dinah.alexander@wits.ac.za (D.A.)

**Keywords:** Attention Deficit Hyperactivity Disorder (ADHD), sibling’s perceptions, parenting differences

## Abstract

This qualitative study explored siblings’ perceptions of the impact a sibling diagnosed with ADHD has within the family system. Specific focus was placed on the different ways these different sibling cohorts were parented. Participants constituted eight adult females with a mean age of 20 years from different cultural and socio-economic backgrounds in the province of Gauteng, South Africa. Data was collected using semi-structured interviews and was analysed thematically. The four themes that emerged from the interviews include differential parental treatment, rejection, discrepancy with discipline, and the parentified child. Specifically, the results of this study revealed feelings of differential parental treatment and discipline that took place in the home and rejection experienced by the non-ADHD sibling. There was also a common theme of a parentified child, who had to carry a burden of caring for their sibling with ADHD. The non-diagnosed siblings perceive themselves to be particularly negatively impacted. The results are discussed in light of the previous empirical studies, and recommendations are made.

## 1. Introduction

Attention deficit hyperactivity disorder (ADHD) is one of the most common and challenging childhood neurobehavioral disorders globally. ADHD is known to negatively impact children, their families, and the community [1,2]. A large body of research conducted in developed Euro-Western countries reveals high levels of family dysfunction in families of children diagnosed with ADHD [3,4,5,6,7]. It has been well established that the manifestation of ADHD behaviours is sufficient to cause impairment or significant disruption in major life activities, such as schooling and social skills [2,8]. Johnston and Mash [9] reviewed a body of literature to examine the effect of having a child with ADHD on family functioning. They concluded that the presence of a child with ADHD results in an increased likelihood of disturbances to family and marital functioning, disrupted parent-child relationships, reduced parenting efficacy, and increased levels of parental stress. 

Having taken the above research into account, the authors of this study aimed at paying particular attention to the non-ADHD sibling in the hopes of promoting positive outcomes for the development and relationships of these individuals and their families.

Although the impacts of ADHD behaviours have been widely reported from the perspectives of children and adolescents diagnosed with ADHD, educators, and from parents’ perspectives [6,10,11,12,13], not much is known about the perceptions of siblings of diagnosed children, particularly in a South African context. As such, this study hoped to provide a voice to those non-ADHD siblings who had first-hand experience as to what it was like living with an ADHD sibling and the possible challenges that may occur as a result. 

It is, however, acknowledged that there are few studies conducted from Euro-Western countries which explore the perspectives of siblings living with children diagnosed with ADHD. The relationships between the siblings may be affected when there is a child with ADHD. Indeed, an earlier study [14] investigated sibling accounts of the experiences associated with having a sibling with ADHD among 43 participants. The results indicated that non-ADHD siblings reported feeling as though the focus of their family was on their sibling with ADHD. Non-ADHD siblings also expressed feelings of victimization, loss, and sorrow, as well as the pressure to care for their sibling due to parental expectations [14]. Harpin [15] found a significant amount of distress and dysfunction among siblings of children diagnosed with ADHD. Salmeron [16] unearthed feelings of victimisation through violent and aggressive behaviours and manipulation among siblings of children diagnosed with ADHD. In another study, Mikami and Pfiffner [17] compared the siblings’ relationships of children diagnosed with ADHD with the control group of siblings living without a diagnosis of ADHD among 91 participants. The results revealed that sibling relationships in the ADHD group had significantly higher levels of conflict than the control group. Listug-Lunde, Zevenbergen, and Petros [18] argue that there is limited research that investigates the impact ADHD can have on siblings, and that for that reason, this area should be explored in more depth.

Given the paucity of research on how a child with ADHD may affect the family ecosystem (especially from the African continent), it is crucial to explore the perceptions of the siblings living with children diagnosed with ADHD in order to understand the impact of their siblings’ behaviours and how it can be managed. It is believed that the more research is conducted on siblings’ perceptions, the more awareness it will generate, and will thus encourage more support from organizations and other social spheres. 

Relationships between siblings and other members of the family may be negatively affected, particularly when there is a child with ADHD. Siblings of ADHD children have reported feeling ill-treated through violent and aggressive behaviours, and manipulation. For instance, jealousy is often experienced by siblings because of the amount of attention given to the ADHD child by their parents [16].

In another study, Mikami and Pfiffner [17] explored the relationships of ADHD children with their siblings and compared them to relative non-problem children (control group) in a sample of 91 children between the ages of 5 and 11 years old. It was hypothesized that sibling relationships among ADHD children would have more conflict and less warmth/closeness; comorbid conditions would be associated with a higher degree of sibling conflict. The results of the study demonstrated significantly higher levels of conflict in the ADHD group than in the control group. In addition, comorbid externalizing problems were associated with less warmth/closeness and increased conflict in the sibling relationship, and largely accounted for the finding that children with ADHD showed greater sibling relationship problems. In a qualitative study of 206 boys and girls between the ages of 7 to 13 years old, Counts, Nigg, Stawicki, Rappley, and Von Eye [19] explored the degree of family adversity in ADHD subtypes and behavioural issues and compared to families without an ADHD child. The results of the study concluded that children with ADHD had greater family adversity than children without a diagnosis.

It is imperative that the African context be explored, given that the majority of the countries in Africa experience a low human development index [20]. Therefore, families and individuals might be denied the help that they need due to a lack of funding, socio-economic status, and their location [21]. Additionally, some families may not be aware of their child’s condition due to a lack of education. This in turn could exacerbate the situation and place further strain on the family’s interrelationships. 

The main research question that drove the study was “what are the perceptions of non-ADHD siblings of the impact of a sibling diagnosed with ADHD on the family system?” This question was unpacked with the following sub-questions:

(1) What is the experience of a sibling living with a sibling diagnosed with a mental disorder such as ADHD?
(2) What are the participants’ perceptions of the relationship they have with the caregiver(s) and the sibling diagnosed with ADHD?
(3) What did the participants observe regarding parenting in the home while living with a sibling with ADHD?

## 2. Materials and Methods

### 2.1. Research Design

This study applied a qualitative method of investigation. Given the nature of the study, a phenomenological research design was deemed appropriate, as participants’ lived experiences were essential [22,23].

### 2.2. Sampling Procedures

The participants were accessed through convenience sampling. Snowball sampling was also used to access additional participants known through word of mouth. Criteria for participation in the study included individuals in early adulthood (between the ages of 18 and 30), with a reasonable degree of fluency in English. The participant’s age was imperative, as the researchers required a comprehensive and in-depth response to the possible sensitive nature of the topics raised. The quality of the data was therefore determined to a large extent by the participant’s ability to accurately recall past exposures. In order to prevent recall bias, participants were blinded to the study hypothesis. Additionally, a requirement was that all the participants had to have resided with a sibling who was diagnosed with ADHD, as well as a caregiver or parent in the same household. The participants were not reimbursed for participating in the study. They were met on university campus at a time and place of their convenience. No formal testing took place to screen the participants for ADHD themselves. The authors relied on the feedback received from the participants, and they acknowledge that while the possibility exists that the participants may have had ADHD symptoms, this is unlikely to have been the case. Furthermore, participants were required to disclose any concerns they had regarding their own behaviour in terms of ADHD.

### 2.3. Participants

The participants in the current study comprised eight females between 18 and 30 years old. While attempts were made to gather perspectives of the male sibling, the research was not successful in this regard. The majority of the participants were aged 18 years (63%, *n* = 5), while the least number of participants, (37%, *n* = 3) were within the older age range of 20–29 years. The overall mean age of the sample was 20 years. Table 1 presents an overview of the participants’ socio-demographic and background information.

### 2.4. Testing and Intervention Procedure

Permission to conduct this research was initially obtained from the University’s Ethics Committee (Non-Medical) (Project Identification Code: MACC/14/002 IH). Permission was also sought from the Heads of the School from the Faculty of Humanities. A message regarding the study was then sent out to the first year psychology students. All participants were briefed of their rights to confidentiality: that they could choose to participate or not to participate in the study, and would not be disadvantaged in any way. Permission to audio-record the interview was also granted by the participants. They were informed that their anonymity would be ensured in the reporting of the results.

### 2.5. Instrument

A semi-structured interview was used for the data collection. Open-ended questions were posed to the participants, some of them being:

(1) Can you tell me what the relationships were like within your family system?
(2) How did you perceive your caregiver/parent response to the diagnosis of your ADHD sibling?
(3) Can you tell me what your role was as a sibling, after the diagnosis of your ADHD sibling?

This study was premised on the assumption that the experiences of the non-ADHD siblings could best be understood through interaction [24]. The interviews focused on the exploration of the participant’s experience with regard to living with a sibling that had been diagnosed with ADHD and the impact thereof if any. The interviews were conducted over a period of one month, and each lasted approximately forty-five minutes to an hour. The interviews were conducted in English. 

### 2.6. Data Analysis

Recorded interviews were transcribed verbatim, and a thematic analysis was conducted to analyse the qualitative data gained from the semi-structured interviews [22,23]. Themes were generated inductively from the raw data through reading and re-reading the transcripts. The recurring themes were identified and then categorized within the data, rather than identifying coded words. This was followed by analysis and interpretation of the participants’ perceptions. This study involved exploring how people make sense of their world and how they interpret and understand certain events and experiences [24]. This study falls within the interpretivist paradigm, as the aim of the research was to understand and explore the perceptions of siblings of ADHD children [24]. 

## 3. Results and Discussion

The results of the present study are presented concurrently with the discussion. The following themes that relate to the non-ADHD sibling’s perceptions of the impact a sibling diagnosed with ADHD has on the family system emerged from the data; namely, differential parental treatment that took place in the home, rejection faced by the non-ADHD sibling, a discrepancy in terms of discipline, and the phenomenon of the parentified child.

### 3.1. Differential Parental Treatment and Its Impact

It emerged from the analysis that there was differential parental treatment in the forms of attention provided to children and inconsistent disciplinary methods at home. The results of the present study demonstrated within the non-ADHD siblings feelings of not getting adequate attention and that their sibling with ADHD “*got more attention*”*.* In addition, resources were provided for their sibling but not for them, despite the fact that they, too, needed extra help. Some of the excerpts indicated that:

“*He got more attention.*”

“*He always got more attention because he wanted more attention.*”

“*Everything revolved around my brother and it almost was like it had to go his way.*”

“*My needs would always be ignored.*”

“*There were times when we were shopping for stuff for me and if my brother got tired or bored they would have to go home. This made me feel jealous in a way and cheated out of a lot of things because he had ADHD and I didn’t. It was unfair to me.*”

Participant 2 felt that she could have benefitted from additional support such as a tutor, but her parents insisted she did the work on her own. However, her brother “*had four different tutors, one for each subject*”. Participant 4 reported that her parents “*would brush off*” what she needed, yet they would do everything for her sister. This in turn affected the relationship between the non-ADHD sibling and the parent(s).

The tendency for parents to inadvertently give more attention to the diagnosed child is consistent with previous studies. Research has revealed reduced parental attention paid to the child without ADHD [25,26]. Similarly, it was found in an earlier study that the non-ADHD siblings reported feeling as though the focus of their family was on their sibling with ADHD [14,16].

### 3.2. Rejection Faced by the Non-ADHD Sibling

The results revealed a consistent theme of rejection resulting from the lack of or absence of attention from the parents because their siblings with ADHD needed the assistance and support, for they were the ones with the disorder. For instance, Participant 8 expressed that when all the attention was on her sister and she was not considered, it made her feel “*a little sad and a bit angry*”*.* Participant 6 stated that she “*felt a bit bitter*” towards her parents for always defending the brother and taking his side. She said that she developed this attitude:

“*Just go help your son; he needs you more than me.*”

Participant 7 reported that her feelings of rejection were so severe that she started to abuse drugs and alcohol in order to fill the void in her life:

“*Because of my addictive personality I became very addicted. I think it is because of all the rejection I felt from my mom.*”

In addition to this, Participant 6 stated that she remembered that:

“*I would just go and hide and cry and stuff. I remember there was this one little cupboard on the floor in my mom’s room that did not have anything in it. This used to be my crying spot. So I would just go and retreat in there.*”

These findings are consistent with previous studies conducted in developed Euro-Western countries. Research indicates that while siblings are likely to vary from one to another, the perception of the absence of parental attention is commonly experienced [14,25,26]. 

### 3.3. A Discrepancy with Discipline

One of the main concerns that were expressed by several of the participants in the current study was the fact that the ADHD child would receive few consequences for bad behaviour when it came to homework and chores. Participants reported that their sibling with ADHD could do things that they were never allowed to do when they were young, purely as a result of the ADHD diagnosis. For example:

“*They are more lenient with her and when she performs or has a tantrum because they are disciplining her, they just take away the discipline and the threats.*”

“*They just take away the discipline.*”

“*…when you punish her you take away again the punishment (sic) whereas with us when we are punished, we are punished. There is no going back.*”

“*They make excuses for the naughty behaviour.*”

“*When I was in Grade 1, I had to do a minimum of an hour of homework every day and even if I did not have homework to do my parents made stuff for me to complete. My brother, on the other hand, would finish only half of the required work and when he did not want to do anymore mom or dad would do the rest for him.*”

Many of the participants expressed that they would speak to their parents about the discrepancy in the way in which they were disciplined and would mention that they could see that there was a significant difference in the way in which they were being treated. However, the parents would often create an excuse or try and justify their own behaviour. This resulted in an angry, irritated, and often frustrated sibling. These results seem to mirror those which demonstrated that the siblings of ADHD children feel victimized and unnoticed by the parents [27]. The participants’ responses are significant in relation to the systems theory that reflects that changes to one part of the system are thought to affect the whole system [28,29]. This can be seen in the way in which the ADHD child behaves and acts within the family system and how they had a profound effect on the rest of the family unit. 

It can then be said that the effects the participants felt negatively impacted the parent-child relationship and altered the child’s perceptions towards the caregiver that was instilling authority. Furthermore, this hindered the non-ADHD individual’s development, as the relationships within different parts of the microsystems were not working together for the good of the child.

### 3.4. The Parentified Child

The last theme that emerged from the analyses involved the parentified child. Most of the non-ADHD siblings reported that their parents expected them to play with and supervise their siblings with ADHD. Amongst the caretaking activities were giving medication and helping with homework, as illustrated by the following excerpts:

“*I took on the motherly role and disciplined my brother. I was always the disciplinarian with him, even now.*”

“*I was the one who took care of things. I was the one who gave her medicine.*”

“*At first I was a little bit annoyed and irritated about it because now I had more responsibility, making sure that she is taking her tablets and stuff like that. And I also helped him with his homework and stuff so, whenever we had to do the homework and helping out with stuff it just took hours.*”

“*I had an obligation to look after my ADHD sibling and do what the parent could not. I had to take her to her room and take care of her in order to protect her sister from the violence in the home.*”

Based on the excerpts above, it can be seen how participants in the current study were involuntarily made to assume the responsibilities of their caregivers by taking care of their siblings diagnosed with ADHD. Earley and Cushway [30] define parentification as a process in which there is an expectation from a parental figure that a child will fulfil a parental role within the family system, and the caregiving roles can either be explicit or implicit. The participants in the present study had to carry a burden of caring for their sibling with ADHD. These findings seem to mirror those conducted in Euro-Western countries, which not only showed that non-ADHD siblings shoulder an unintended responsibility of tending for their siblings, but also reveal feelings of resentment that may occur as a result of role reversals [14,30].

With this being said, it goes against the pivotal point that Bronfenbrenner [31] so clearly demonstrates in all his writings. He believes that the primary relationship should ideally be fostered by a person that is within the child’s immediate sphere and is one that involves a complexity of interaction. He further states that this should ideally be provided by primary adults such as the parent or immediate caregiver [32]. In the South African context today, this is not always feasible, due to HIV, parents working long hours, death of family members, and absent parents. Atilola [33] suggested that due to these challenges particular to the South African context and specifically related to “at risk” communities, children might not find the affirmation that should be present in the child-parent (or child/other important adult) relationship, and might consequently search for it in alternate places [33]. This might include the use of drugs or alcohol and/or other risk-taking behaviours.

## 4. Limitations, Strengths, and Recommendations

Like any other study, it is important to acknowledge the limitations of this study. Obtaining information across ecological levels is logistically complex [34], and was beyond the scope of this study. A major limitation of this study was the small sample size, which may not be truly representative of every sibling living with a sibling who has been diagnosed with ADHD. 

However, the fact that this current study focused on sibling’s perceptions of living in a home with an ADHD sibling, within a South African context, suggests that this study is highly relevant due to the high prevalence of children diagnosed with ADHD in South Africa [12]. The research can be furthered by exploring the implications for the parents’ own parenting and the education they receive, as this might have an impact on the way in which they parent their own children. Due to the limited research on ADHD in South Africa, it might be beneficial to extend such research, especially due to the high prevalence of the diagnosis of ADHD and its consequent impact on the family system. Thus, a larger sample size would also be recommended in order to gain greater insight into how the non-ADHD sibling perceives their experience in the home, living with a sibling diagnosed with ADHD. This would allow for a more coherent and in-depth perspective on the topic as well as incorporating variety into the study. This recommendation is formed due to the limitation that only females were interviewed.

Furthermore, it is recommended that a larger study with participants from different socio-economic backgrounds be conducted. The fact that the participants are from middle to upper social-economic background may limit generalisation of the results to the population of South Africa. Nonetheless, they are still applicable to the upper-class population.

Another major limitation to this study is the fact that only females were interviewed. While the authors did try and include males, two of the participants interested had ADHD and had to be excluded from the study. The female participants that did take part in this study may have viewed their roles quite differently from a male subject. Thus, one has to be cautious when interpreting the results and making sweeping statements or generalisations. 

Additionally, it is recommended that increased support be offered to family members who have a child that is diagnosed with ADHD. This might be in the form of classes, support groups, and information booklets. This is due to the fact that once a child is diagnosed with ADHD, it is not only the child that needs treatment, but the whole family should receive counselling and education on issues such as those raised in this study. This may include parenting skills appropriate to the situation and inculcating an awareness in the parents of possible effects on the ADHD child’s siblings. 

## 5. Conclusions

The findings of the research therefore signify that the ADHD sibling does indeed have an impact on the family system, and these impacts are consistent with those reported in the literature. The four themes that emerged from the interviews—namely, differential parental treatment, rejection, discrepancy with discipline, and the parentified child—confirmed that the non-ADHD siblings perceived a difference in the way in which they were treated by their parents, compared to their ADHD sibling. Additionally, it was evident from the way in which the participants articulated their concerns that they perceived themselves to be a hindrance to their parents, because their ADHD siblings needed the assistance and support, as they were the ones with the disorder. The perception was thus rejection. Furthermore, in terms of discipline, all the participants felt that there was a discrepancy, as the ADHD sibling faced fewer consequences and punishment.

The non-ADHD siblings reported that they felt they had a particular role to play in terms of caring for their sibling with ADHD, and many felt it was their responsibility to ensure that their sibling was attended to. However, despite the challenges that these participants faced, most of them found a way to deal with their sibling with ADHD and accommodate their needs.

## Figures and Tables

**Table 1:** Participants’ socio-demographic information.

Participant	1	2	3	4	5	6	7	8
Age	18	18	28	23	22	18	18	18
Gender (M/F)	F	F	F	F	F	F	F	F
Race	White	African	White	White	African	Indian	White	Indian
Lives at home	Yes	Yes	Yes	Yes	Yes	Yes	Yes	Yes
Does the ADHD sibling still live at home	Yes	No	Yes	Yes	Yes	Yes	Sometimes: divorced parents	Yes
Socio-economic status	Upper class	Middle class	Middle class	Upper class	Middle class	Upper class	Middle class	Upper class
Number of siblings	1	4	1	2	3	1	1	2
Sex of sibling with ADHD	F	F	M	M	M	M	M	F
Age of participant when sibling was diagnosed	13	Primary school	20	Don’t know	18	7 or 8	7	15
Age of sibling when diagnosed with ADHD	10	4 or 5	16	8 or 9	6	4	5	13
Parent diagnosed with ADHD	No	Yes (mother)	No	No	No	No	Mom has symptoms	No
Treatment/counselling received	No	Yes	No	No	No	Not sure	Read books	No
Medication for ADHD	Yes	Yes	Yes	Yes	Yes	Yes	Yes	Yes
Behaviour before medication	Easily irritated, impulsive, easily distracted	Impulsive, didn’t listen, could not focus or concentrate	Easily distracted, needed structure, impulsive	Hyperactive, demanding, moody, constant need of attention	Hyperactive, didn’t listen, couldn’t focus, tiresome	Bullying behaviour, impulsive, irritating and destructive, demanding of attention	Demanded attention, hyperactive, impulsive	Demanded attention from her parents, constantly busy, restless and bubbly
Behaviour after medication	Placid and subdued	Placid, less chaotic, quiet and reserved	More controllable, focused	Could concentrate, less hyperactive	More manageable and controlled, calm and focused	Participant stated that her brother got naughtier	Participant 7 stated that the medication numbed her brother and didn’t let him feel anything	Very quiet and subdued, her personality changed
